# Chromatinopathies: clinically overlapping disorders, revealing novel variants and their DNA methylation signatures

**DOI:** 10.1186/s13148-026-02120-1

**Published:** 2026-04-09

**Authors:** Asuman Koparir, Jennifer Kerkhof, Jessica Rzasa, Eva Metzger, Paulina Bahena Carbajal, Konstantinos Kolokotronis, Erkan Koparir, Yvonne Jelting, Michaela A. H. Hofrichter, Jörg Klepper, Thomas König, Eva Runkel, Wahyu Eka Prastyo, Jonas Deinlein, Neda Dragicevic Babic, Juliane Spiegler, Nicole Stachelscheid, Erdmute Kunstmann, Thomas Haaf, Bekim Sadikovic, Eva Klopocki

**Affiliations:** 1 Institute of Human Genetics, Julius Maximilians University Würzburg, Würzburg, Germany; 2 Verspeeten Clinical Genome Centre, London Health Sciences Centre, London, ON Canada; 3 MVZ Genetikum GmbH, Neu-Ulm, Germany; 4 Institute of Medical Genetics, University Hospital Rostock, Rostock, Germany; 5 Institute of Medical Genetics, University of Zurich, Zurich, Swiss Confederation Switzerland; 6 Center of Human Genetics, Tübingen, Germany; 7 Department of Pediatrics and Neuropediatrics, Klinikum Aschaffenburg-Alzenau, Aschaffenburg, Germany; 8 Department of Pediatrics, University Hospital Würzburg, Würzburg, Germany; 9 Present Address: Institute of Clinical Genetics and Genomic Medicine, University Hospital Würzburg, Würzburg, Germany; 10 Department of Pathology and Laboratory Medicine, Western University, London, ON Canada

## Abstract

**Background:**

Chromatinopathies represent a genetically and clinically heterogeneous group of neurodevelopmental disorders (NDDs) caused by pathogenic variants in genes regulating chromatin structure and function. The phenotypic overlap and genetic complexity of these conditions pose significant diagnostic challenges, often resulting in unresolved variants of uncertain significance (VUS).

**Results:**

We investigated a cohort of 400 routine diagnostic individuals with NDD using whole-exome sequencing and genome-wide DNA methylation profiling via the clinically validated EpiSign assay. Episignature classification was used to aid in variant interpretation and to define molecular subtypes of chromatinopathy. Seventeen percent of individuals (67/400) harbored variants in chromatinopathy-associated genes, including 55 novel variants. DNA methylation profiling was performed in 60 individuals with 62 variants in chromatin regulator genes. Of these, 26 individuals (43%) exhibited disorder-specific episignatures consistent with the associated clinical diagnosis. Importantly, methylation profiles supported the pathogenicity of several variants previously classified as VUS and demonstrated diagnostic concordance with known disease-associated genes including *ANKRD11, SETD5, KMT2A, KDM5C, CHD8*, and others.

**Conclusion:**

Our study highlights the improved diagnostic yield and clinical utility of combining genomic and epigenomic profiling in patients with clinically and/or genetically suspected chromatinopathies. Integration of EpiSign analysis facilitated variant reclassification, delineated genotype-epigenotype-phenotype correlations, and expanded the episignature atlas for rare neurodevelopmental disorders.

**Supplementary Information:**

The online version contains supplementary material available at 10.1186/s13148-026-02120-1.

## Background

Next generation sequencing (NGS) technologies allow elucidation of the genetic basis of neurodevelopmental delays and enable precise diagnostics, resulting in chromatinopathies emerging as one of the most rapidly expanding groups. Chromatinopathies are a class of NDDs caused by mutations affecting different chromatin regulators and comprise more than 100 disorders, which are individually rare conditions but collectively represent a relatively common cause of NDDs [1, 2]. Patients affected by chromatinopathies display shared clinical features, developmental delay, intellectual disability, facial dysmorphism, and behavioral disturbances. Interestingly, the phenotypic overlap between these syndromes appears to reflect the molecular interaction of chromatin modifiers, which act via common cellular mechanisms and pathways [3]. Cornelia de Lange syndrome (CdLS, MIM #122,470), Rubinstein-Taybi syndrome (RSTS, MIM #180,849), KBG syndrome (KBGS, MIM#148,050), Coffin-Siris syndrome (CSS, MIM #135,900), and Wiedemann-Steiner syndrome (WDSTS, MIM#605,130) are prominent examples of chromatinopathies.

Recent advances in clinical epigenomics have established DNA methylation episignatures as robust and highly specific biomarkers for a growing number of rare neurodevelopmental disorders. Episignatures represent disease-specific-methylation patterns in peripheral blood, which are particularly valuable in cases where genomic testing yielded inconclusive results. The EpiSign assay—a clinically validated genome-wide DNA methylation profiling platform—enables detection of over 100 disease-associated episignature and has demonstrated utility in confirming molecular diagnoses, interpreting VUS, and screening patients with complex phenotypes [4–7]. Integration of methylation-based classification with genomic data enhances diagnostic confidence and can identify epigenomic correlates of functional pathogenicity that are not apparent through sequence analysis alone. This combinatorial approach has been shown to improve clinical resolution and reduce diagnostic odyssey in rare disease populations [8].

In this study, we analyzed 400 routine diagnostic cases with NDD and additional clinical features. We performed whole-exome sequencing to identify the underlying molecular pathology. In addition, genome-wide DNA methylation data were obtained from individuals harboring variants in chromatinopathy-associated genes and analyzed against the full EpiSign V5 classifier menu, encompassing episignatures associated with both chromatinopathy and non-chromatinopathy genes. Nevertheless, the analytical focus of the manuscript was placed on individuals harboring variants in chromatinopathy-associated genes. The decision to focus on this specific cohort was driven by biological and clinical considerations rather than methodological constraints.

Chromatinopathies constitute a mechanistically coherent class of disorders in which altered chromatin structure and epigenetic regulation play a central role in disease pathogenesis. Consistent with prior reports, disorders affecting chromatin regulation are more likely to exhibit strong and reproducible episignatures, making this group particularly well suited for for assessing clinical DNA-methylation based biomarker utility. Sixty-seven (17%) of our patients with NDD demonstrated 66 different variants in 39 chromatinopathy genes. A genome-wide DNA methylation profile characteristic of chromatinopathies allowed functional classification of these variants. Therefore, we assessed genome-wide DNA methylation profiles in a cohort of 60 patients with variants in chromatinopathy genes.

## Materials and methods

In this study, 400 individuals with NDD and additional clinical features were collected from clinical genetic diagnostics at the Human Genetics Institute of Würzburg University between 2018 and 2023. Most of our patients had previously tested negative for molecular karyotyping and *FMR1* CGG expansion. We performed whole-exome sequencing as a diagnostic tool to identify the underlying molecular pathology. In parallel with whole-exome sequencing (WES), Competitive Allele-Specific PCR (CASP) assays were systematically performed across all cases to evaluate potential sample mix-ups. The results definitively ruled out cross-contamination and sample misidentification among the collected blood specimens. Copy number variants (CNVs) and structural variants (SVs) were evaluated using WES-based analytical tools; however, this approach does not provide comprehensive genome-wide coverage regarding larger CNVs, SVs, deep intronic variants, or complex structural rearrangements.

### WES and variant classification

Exome capture was performed according to the Illumina Nextera Rapid Capture Enrichment library preparation protocol using 50 ng of genomic DNA from patients. Paired-end sequencing of the libraries was performed with a NextSeq2000 and NextSeq500 sequencer and the v2 reagent kit (Illumina, San Diego, California, USA). Sequences were mapped to the human genome reference (NCBI build 38/hg38 version) using the Burrows-Wheeler Transform and obtaining a mean coverage of ≥ 50X in approximately 91% of the target regions. Approximately 99% of the exome was covered at least 10X. Variants were called and analyzed using GensearchNGS software (PhenoSystems SA, Braine le Chateau, Belgium). Variants with a coverage of ≤ 10X, a Phred-scaled quality of ≤ 15, a frequency of ≤ 15%, and a MAF of ≥ 2% were neglected. Alamut Visual (Interactive Biosoftware, Rouen, France) software including prediction tools like SIFT [9], MutationTaster [10], PolyPhen-2 [11], CADD [12], and REVEL [13] was used for variant prioritization. Potential effects of a variant on pre-mRNA splicing were evaluated by SpliceAI [14], SpliceSiteFinder-like, MaxEntScan, NNSPLICE, GeneSplicer, Human Splicing Finder, ESEfinder, RESCUE-ESE, and EX-SKIP. Population databases like gnomAD v4.1.0 [15] revealed whether a variant has been previously found. Variants were queried through the Human Gene Mutation Database (HGMD [16]), ClinVar and the Leiden Open Variation Database (LOVD). The compound heterozygous variants were validated by segregation analysis in the index patients and available family members using Sanger sequencing on an ABI 3130xl 16-capillary sequencer (Life Technologies). Primer sequences were designed using Primer 3 [17]. Primer sequences are available upon request. Variant classification followed the ACMG/AMP criteria established by Richards et al. [18]. In addition to Richards et al., the updated criteria from the following publications were used: ClinGen Sequence Variant Interpretation Recommendation for application of PM2 and PM3, Abou Tayoun et al. (2018) for application of PVS1 [19], Brnich et al. (2020) for application of PS3 [20], Pejaver et al. (2022) for missense variant classification [21], and Walker et al. (2023) for splice variant classification [22].

### DNA methylation episignature analysis

Methylation analysis was conducted using the clinically validated EpiSign V5 assay, following previously established methods [5, 7, 8, 23]. EpiSign V5 test menu and caveats outlined in supplementary Table 2. Methylated and unmethylated signal intensities generated from the EPIC V2 arrays were imported into R 4.2.1 for normalization, background correction, and filtering. Beta values were then calculated as a measure of methylation level, ranging from 0 (no methylation) to 1 (complete methylation), and processed through the established support vector machine (SVM) classification algorithm for EpiSign disorders. The classifier utilized the EpiSign Knowledge Database, which consists of over 20,000 methylation profiles from reference disorder-specific and unaffected control cohorts, to generate disorder-specific methylation variant pathogenicity (MVP) scores. These MVP scores are a measure of prediction confidence for each disorder and range from 0 (discordant) to 1 (highly concordant). The reference database used for episignature analysis comprises approximately 20,000 DNA methylation profiles. The majority of these profiles are derived from healthy individuals, while a subset corresponds to patients with known specific disorders. The number of profiles available for each disorder varies, which can influence the sensitivity and predictive accuracy of episignature detection. The final EpiSign result is a combination of the three assessed parameters: MVP score, Euclidean clustering, and multidimensional scaling plots. The result is reported with a confidence level relative to the reference episignature cohorts, where high confidence indicates agreement among all three parameters and moderate confidence indicates disagreement in at least one of the three.

## Results

### WES revealed variants in chromatinopathy genes

**Fig. 1:**
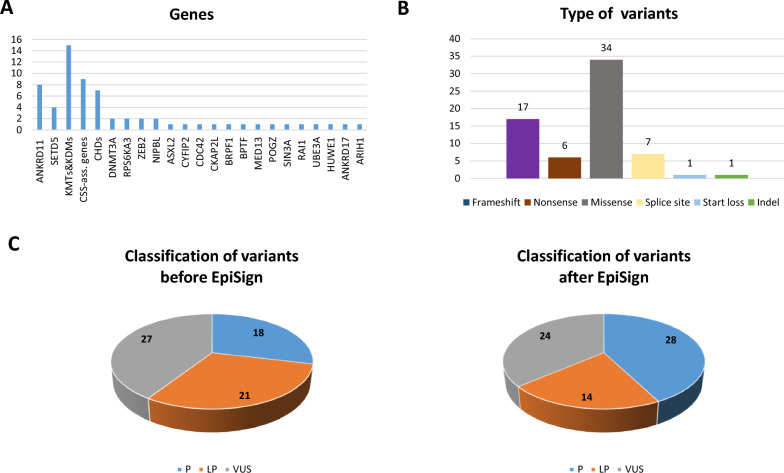
Distribution of identified variants by gene, variant type, and classification. **A** Chromatinopathy genes with the number of detected variants. **B** Type of detected variants. **C** Classification of detected variants according to ACMG/AMP criteria before and after episignature analysis

We detected 205 variants in our 400 NDD patients. Thirty-two percent (n = 66) of all detected 205 variants were in chromatinopathy genes**.** Variants in *ANKRD11* (n = 8) and *SETD5* (n = 4) were the most common within chromatinopathy genes. We detected eight variants in *ANKRD11* and four variants in *SETD5*. Of these eight (six in *ANKRD11*, two in *SETD5*) variants are truncating (c.4554del, c.3702_3705del, c.6812_6813del, c.3180dup, c.7234C > T, c.6349_6362del in *ANKRD11* and c.2182del, c.1333C > T in *SETD5*). Twenty-four percent (n = 16) of our patients exhibited variants in the histone lysine methylases/ histone demethylases (*KMT/ KDM*) genes: *KMT2A* (n = 4), *KDM5C* (n = 6), *KMT2D* (n = 2), *KDM5B* (n = 1), *KMT2B* (n = 1), *KMT2C* (n = 1), and KMT2E (n = 1). We identified nine variants in CSS causative genes including seven VUSs in: *ARID1A* (c.6416C > A)*, ARID2* (c.5030G > A), *BICRA* (c.3218C > G), *SMARCD1* (c.217G > A)*, SMARCA4* (c.761G > T, c.2118C > G, c.3919G > A), a truncating variant in *ARID2* (c.1109dupT) and an essential splice-site variant in *SMARCB1* (c.363-2A>G). Six different CHD variants were identified in seven patients: *CHD3* (c.3692G > A, c.3736C > T), *CHD4* (c.4213del, c.1021C > T), *CHD5* (c.870 + 6 T > C), and *CHD8* (c.4728-2A > G). Three of these variants were classified as likely pathogenic or pathogenic according to the ACMG/AMP criteria, whereas the other three were classified as VUS. The remaining 21 patients had 24 different variants in the following genes (Table 1, Fig. 1A): *ACTL6A, ANKRD17, ARIH1, ASXL2, BPTF, BRPF1, CDC42, CKAP2L, CREBBP*, *CYFIP2, DNMT3A*, *HUWE1, MED13, NIPBL, POGZ, RAI1, RPS6KA3, SIN3A, UBE3A* and* ZEB2.*

**Table 1:** Genetic landscape of cases in this study

P-ID	*Gene*	Transcript	Variant	AA change	Zygosity	Inheritance	Classification°	Reference	EpiSign V5
P1	*ACTL6A*	NM_004301.4	c.1166G > A	p.(Arg389Gln)	het	NA	VUS	This study	None*
P2	*ANKRD11*	NM_013275.5	c.4554del	p.(Arg1519Glyfs*12)	het	de novo	P	This study	None
P3	*ANKRD11*	NM_013275.5	c.3702_3705del	p.(Lys1235Argfs*82)	het	de novo	P	This study	KBGS_MRD23
P4	*ANKRD11*	NM_013275.5	c.7535G > A	p.(Arg2512Gln)	het	de novo	P	[44]	Moderate KBGS_MRD23
P5	*ANKRD11*	NM_013275.5	c.6812_6813del	p.(Pro2271Argfs*24)	het	Maternal	P	[45]	KBGS_MRD23
P6	*ANKRD11*	NM_013275.5	c.3180dup	p.(Asp1061Argfs*7)	het	NA	P	This study	KBGS_MRD23
P7	*ANKRD11*	NM_013275.5	c.800G > A	p.(Gly267Asp)	het	NA	VUS	This study	None
P8	*ANKRD11*	NM_013275.5	c.7234C > T	p.(Gln2412*)	het	de novo	P	This study	KBGS_MRD23
P9	*ANKRD11*	NM_013275.5	c.6349_6362del	p.(Pro2117Glyfs*25)	het	de novo	P	This study	KBGS_MRD23
P10	*ANKRD17*	NM_001286771.3	c.1616A > C	p.(Lys531Thr)		NA	VUS	This study	None*
P11	*ARID1A*	NM_006015.6	c.6416C > A	p. (Pro2139His)	het	de novo	VUS	This study	None
P12	*ARID2*	NM_152641.3	c.5030G > A	p.(Arg1677Gln)	het	NA	VUS	This study	Inconclusive CSS6
P13	*ARID2*	NM_152641.3	c.1109dupT	p.(Leu370Phefs*18)	het	NA	P	This study	CSS6
P14	*ARIH1*	NM_005744.5	c.1285C > T	p.(Arg429Cys)	het	NA	VUS	This study	None*
P15	*ASXL2*	NM_018263.6	c.3384T > G	p.(Ile1128Met)	het	NA	VUS	This study	None*
P16	*BICRA*	NM_015711.3	c.3218C > G	p.(Pro1073Arg)	het	NA	VUS	This study	None*
P17	*BPTF*	NM_004459.6	c.613 + 1del	p.?	het	de novo	LP	This study	Inconclusive MRD21*
P18	*BRPF1*	NM_001003694.1	c.2972T > G	p.(Phe991Cys)	het	de novo	LP	This study	None*
P19	*CDC42*	NM_001791.4	c.101C > T	p.(Pro34Leu)	het	NA	LP	This study	None*
P20	*CHD3*	NM_001005271.2	c.3692G > A	p.(Arg1231Gln)	het	de novo	LP	This study	Inconclusive ARTHS*
P21	*CHD3*	NM_001005271.2	c.3736C > T	p.(Arg1246Cys)	het	NA	VUS	This study	None*
P22	*CHD4*	NM_001273.3	c.4213del	p. (Ala1405Profs*41)	het	de novo	P	This study	None
P23	*CHD4*	NM_001273.3	c.1021C > T	p.(Arg341Cys)	het	paternal	VUS	This study	None
P24	*CHD5*	NM_015557.3	c.870 + 6T > C	p.?	het	NA	VUS	This study	None*
P25 sister of P24	*CHD5*	NM_015557.3	c.870 + 6T > C	p.?	het	NA	VUS	This study	Not performed
P26	*CHD8*	NM_001170629.1	c.4728-2A > G	p.?	het	NA	P	This study	IDDAM
P27	*CKAP2L*	NM_152515.4	c.1463_1467del	p.(Thr488Lysfs*16)	homo		P	This study	None*
P28	*CREBBP*	NM_004380.3	c.4186C > G	p.(Leu1396Val)	het	NA	VUS	This study	None
P29	*CYFIP2*	NM_001037333.3	c.260G > A	p.(Arg87His)	het	NA	P	[46]	None*
P2	*DNMT3A*	NM_175629.2	c.427C > T	p.(Arg143*)	het	de novo	P	[41]	None
P30	*DNMT3A*	NM_175629.2	c.897A > C	p.(Lys299Asn)	het	de novo	P	This study	TBRS
P31	*HUWE1*	NM_031407.7	c.1712C > T	p.(Ser571Leu)	het	NA	VUS	This study	None*
P67	*KDM5B*	NM_006618.5	c.406-2A > T	p.?	het	NA	LP	This study	None*
P32	*KDM5C*	NM_004187.5	c.228 + 1G > A	p.?	hem	NA	P	This study	MRXSCJ
P33 brother of P32	*KDM5C*	NM_004187.5	c.228 + 1G > A	p.?	hem	NA	P	This study	Not performed
P34	*KDM5C*	NM_004187.7	c. 1646 T > C	p.(Met549Thr)	hem	de novo	LP	This study	MRXSCJ
P35	*KDM5C*	NM_004187.6	c.73del	p.(Arg25Glufs*48)	hem	Maternal	P	This study	MRXSCJ
P36 mother of P35	*KDM5C*	NM_004187.6	c.73del	p.(Arg25Glufs*48)	het	NA	P	This study	Not performed
P68	*KDM5C*	NM_004187.6	c.1837G > A	p.(Glu613Lys)	het	de novo	LP	[47]	MRXSCJ
P37	*KMT2A*	NM_001197104.2	c.7187del	p.(Pro2396Glnfs*10)	het	de novo	P	This study	WDSTS
P38	*KMT2A*	NM_001197104.2	c.839_843del	p. (Pro280Glnfs*3)	het	NA	P	[48]	WDSTS
P39	*KMT2A*	NM_001197104.2	c.4432_4434del	p.(Arg1478del)	het	NA	P	[49]	WDSTS
P40	*KMT2A*	NM_001197104.2	c.3452G > A	p.(Arg1151Gln)	het	de novo	P	[50]	WDSTS
P41	*KMT2B*	NM_014727.3	c.918G > C	p.(Lys306Asn)	het	NA	VUS	This study	None
P41	*KMT2B*	NM_014727.3	c.3107C > T	p.(Pro1036Leu)	het	NA	VUS	This study	None
P43	*KMT2C*	NM_170606.3	c.4652A > G	p.(His1551Arg)	het	NA	VUS	This study	None*
P44	*KMT2D*	NM_003482.4	c.14527_14528del	p.(Lys4843Glyfs*12)	het	NA	P	This study	Kabuki
P45	*KMT2D*	NM_003482.5	c.11128G > T	p.(Gly3710*)	het	de novo	P	This study	Kabuki
P46	*KMT2E*	NM_182931.3	c.-4_5delinsTTTAC	p.Met1?,	het	NA	VUS	This study	None*
P47	*MED13*	NM_005121.3	c.5377G > T	p.(Gly1793*)	het	NA	LP	This study	None*
P48	*NIPBL*	NM_133433.3	c.3525del	p.(Glu1176Lysfs*13)	het	NA	LP	This study	Not performed
P49	*NIPBL*	NM_133433.3	c.6065A > G	p.(Lys2022Arg)	het	NA	VUS	This study	None
P50	*POGZ*	NM_015100.4	c.2569A > G	p.(Arg857Gly)	het	de novo	P	This study	Moderate WHSUS
P51	*RAI1*	NM_030665.3	c.707del	p.(Tyr236Leufs*16)	het	NA	LP	This study	None*
P52	*RPS6KA3*	NM_004586.3	c.295A > G	p.(Met99Val)	hem	de novo	LP	This study	Not performed
P53	*RPS6KA3*	NM_004586.3	c.1741A > G	p.(Thr581Ala)	hem	de novo	LP	This study	Not performed
P54	*SETD5*	NM_001080517.2	c.1077 + 4A > G	p.?	het	de novo	VUS	This study	None
P55	*SETD5*	NM_001080517.2	c.2182del	p.(Asp728Ilefs*9)	het	de novo	P	This study	KBGS_MRD23
P56	*SETD5*	NM_001080517.2	c.1333C > T	p.(Arg445*)	het	de novo	P	[51]	KBGS_MRD23
P57	*SETD5*	NM_001080517.2	c.1495G > T	p.(Asp499Tyr)	het	NA	VUS	This study	None
P58	*SIN3A*	NM_001145358.2	c.3418C > T	p.(Arg1140*)	het	de novo	P	This study	WITKOS
P59	*SMARCA4*	NM_001128849.3	c.761G > T	p.(Gly254Val)	het	NA	VUS	This study	None
P60	*SMARCA4*	NM_001128849.3	c.2118C > G	p.(Ile706Met)	het	paternal	VUS	This study	None
P61	*SMARCA4*	NM_001128849.3	c.3919G > A	p.(Ala1307Thr)	het	NA	VUS	This study	None
P62	*SMARCB1*	NM_003073.5	c.363-2A>G	p.?	het	de novo	P	This study	BAFopathy
P63	*SMARCD1*	NM_003076.4	c.217G > A	p. (Gly73Arg)	het	NA	VUS	This study	None*
P64	*UBE3A*	NM_130838.2	c.2507_2510del	p.(Lys836Argfs*4)	het	NA	P	[52]	None*
P65	*ZEB2*	NM_014795.4	c.887A > G	p.(His296Arg)	het	NA	VUS	This study	Moderate MOWS
P66	*ZEB2*	NM_014795.4	c.3215del	p.(Gln1072Argfs*3)	het	NA	LP	This study	Not performed

P, pathogenic; LP, likely pathogenic; VUS, variant of unknown significance; het, heterozygous; hem, hemizygous; hom, homozygous; NA, not available; KBGS, KBG syndrome; MRD23, Intellectual developmental disorder, autosomal dominant 23; CSS6, Coffin-Siris syndrome Type 6; MRD21, Intellectual developmental disorder, autosomal dominant 21; ARTHS, Arboleda-Tham syndrome; IDDAM, intellectual developmental disorder with autism and macrocephaly; TBRS, Tatton-Brown-Rahman syndrome; MRXSCJ, Claes-Jensen type of X-linked syndromic intellectual developmental disorder; WDSTS, Wiedemann-Steiner syndrome; WHSUS, White-Sutton syndrome; WITKOS, Witteveen-Kolk syndrome. °The ACMG criteria used for each variant are shown in Table S2. *Samples evaluated on EpiSign V5 screen but no established specific episignature for the underlying gene on EpiSign V5 test menu

In total more than half of these variants 51% (n = 34) belong to the variant type “missense” (Fig. 1B). Fifty-nine percent (n = 39) of all variants are likely pathogenic (LP, n = 21) or pathogenic (P, n = 18). The remaining 27 variants are VUS, almost all of which are missense variants (Table 1, Fig. 1B, C). Following episignature analysis 10 of the 21 variants initially classified as LP were reclassified as P. Subsequently, among the 27 VUS, three were reclassified as LP. Resulting in a final classification of 28 pathogenic, 14 likely pathogenic, and 24 VUS variants (Fig. 1C).

### Genome-wide DNA methylation episignatures

We conducted genome-wide methylation analysis using the EpiSign platform for 60 patients with 62 different variants in chromatinopathy genes. This cohort included patients carrying variants in genes without (n = 21) and with (n = 39) a known episignature. For genes without an established episignature (*ACTL6A, ANKRD17, ARIH1, ASXL2, BICRA, BPTF, BRPF1, CDC42, CHD3, CHD5, CKAP2L, CYFIP2, HUWE1, KDM5B, KMT2C, KMT2E, MED13, RAI1, SMARCD1, and UBE3A*), patients were not assessed as positive or negative. Instead, an unbiased screen against the EpiSign V5 reference database was performed to determine whether the patient’s DNA methylation profile matches any known episignature associated with a different gene. This approach allows exploration of potential shared molecular mechanisms or overlapping epigenetic dysregulation, and may suggest convergent disease pathways or alternative diagnoses, though a match does not confirm pathogenicity for the gene of interest.

**Fig. 2:**
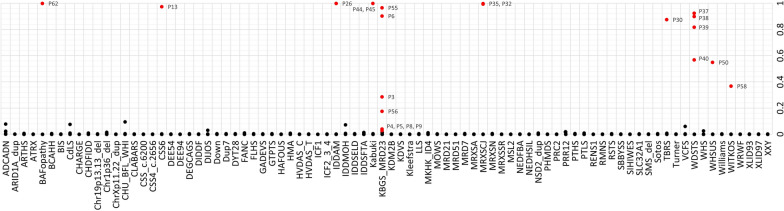
Summary of MVP classification scores for twenty-three likely pathogenic and pathogenic cases with variants in genes associated with defined episignatures on EpiSign V5. MVP is a multi-class supervised classification system capable of distinguishing between multiple episignatures by generating a probability score for each. Each case receives scores across all episignatures, reflecting the likelihood of a match. These MVP scores are shown on the y-axis range from 0 (discordant) to 1 (highly concordant). Positive calls—determined in combination with additional parameters, Euclidean clustering and multidimensional scaling—are highlighted in red

Notably, two patients showed partial overlap with existing episignatures: P17, carrying a *BPTF* variant, partially overlapped with the MRD21 signature, and P20, carrying a *CHD3* variant, partially overlapped with the ARTHS signature (Table 1). These observations likely reflect that a gene-specific episignature has not yet been established, preventing definitive classifier-based interpretation (Fig. 2).

**Fig. 3:**
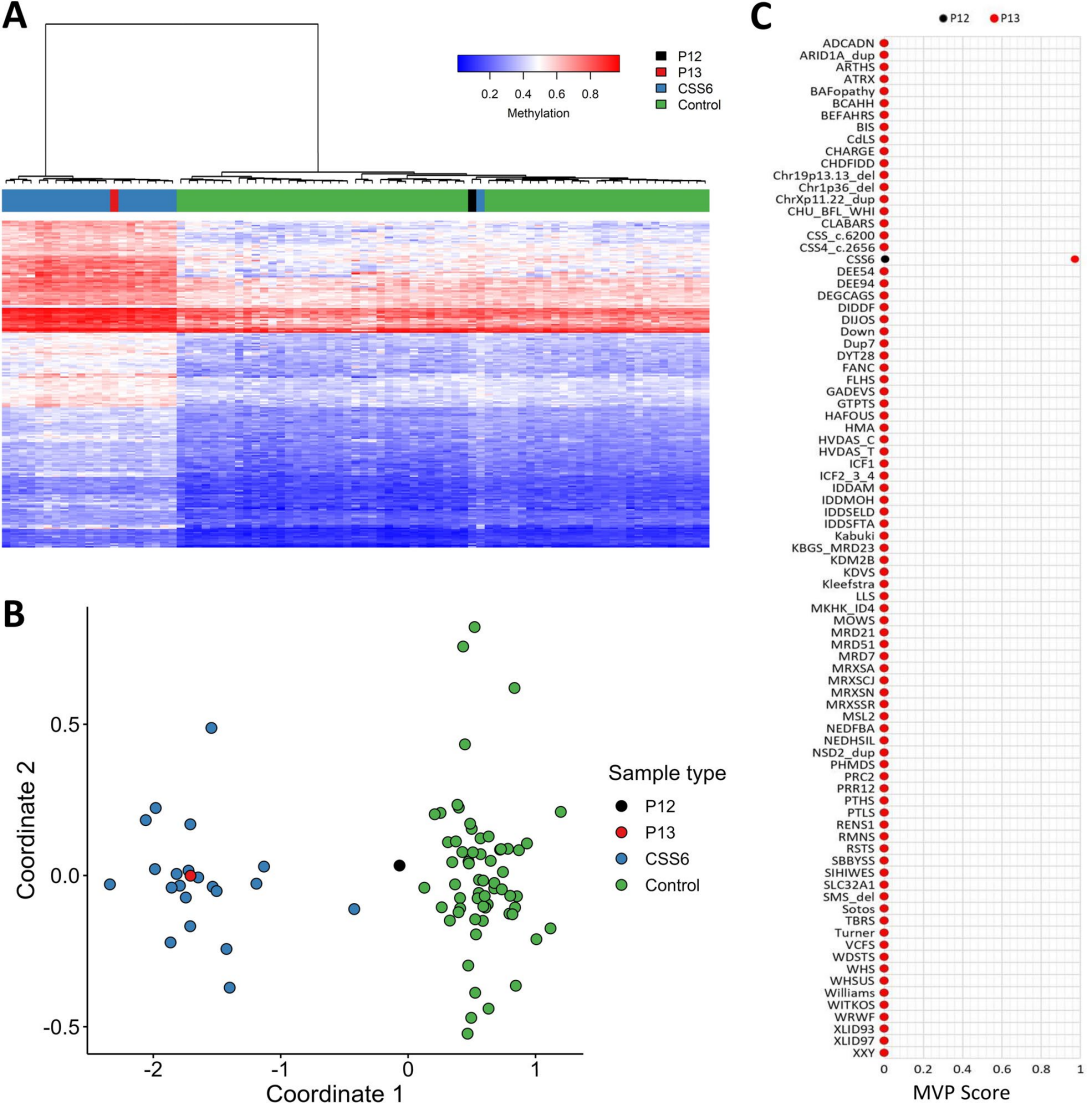
EpiSign (DNA methylation) analysis of peripheral blood from two cases with a VUS missense or LP truncating *ARID2* variant, the causative gene for Coffin Siris Syndrome 6 (CSS6). Hierarchical clustering (**A**) with each row representing a probe and each column a sample and multidimensional scaling plot (**B**) where pairwise distance between points is representative of the similarity between them. P13 (red) has a DNA methylation profile similar to subjects with a confirmed CSS6 episignature (blue) and is distinct from controls (green). P12 (black) shows an intermediate profile which differs from controls (green) but does not exactly match CSS6 cases (blue). **C** MVP score, a multi-class supervised classification system capable of discerning between multiple episignatures by generating a probability score for each episignature. MVP scores are a measure of prediction confidence for each disorder and range from 0 (discordant) to 1 (highly concordant). The elevated MVP score for CSS6 indicates a methylation profile similar to the reference cohort for P13 (0.974)

23 patients carried a single pathogenic or likely pathogenic variant in a gene with a known episignature: BAFopathy (n = 1), CSS6 (n = 1) (Fig. 3), IDDAM (n = 1), Kabuki (n = 2), KBGS_MRD23 (n = 8), MOWS (n = 1), MRXSCJ (n = 2), SIHIWES (n = 1), WHSUS (n = 1), TBRS (n = 1), WDSTS (n = 4), and WITKOS (n = 1) All but one individual tested positive on EpiSign with moderate or high confidence, as indicated by elevated MVP scores(Fig. 2). P22, with a *CHD4* frameshift variant (c.4213del, p.(Ala1405Profs*41), was reported negative for the associated Sifrim-Hitz-Weiss syndrome (SIHIWES, MIM#617,159) episignature. As an example of a high confidence positive Episign test the results of P13 (pathogenic *ARID2* variant) consistent with the CSS6 episignature are illustrated in Fig. 3.

One case, P2, carried two pathogenic variants; one in *ANKRD11* and one in *DNMT3A*, associated with KBGS and Tatton-Brown-Rahman syndrome (TBRS, MIM#615,879), respectively. The genome-wide methylation profile in this case matches none of the associated episignatures (Fig. S1), despite a phenotypic presentation overlapping both syndromes. This case was processed once, and the methylation data was used to predict the patient’s age and sex, both of which matched the expected values (Table S1).

The 15 remaining patients carried 16 different VUS in a gene with an established episignature: BAFopathy (n = 4), CdLS (n = 1), CSS6 (n = 1), KBGS_MRD23 (n = 3), KMT2B (n = 1), MOWS (n = 1), MRXSCJ (n = 2), RSTS (n = 1), and SIHIWES (n = 1); 11 of them tested negative regarding established episignature profiles using EpiSign V5 (Table 1). P12 carries a VUS in *ARID*2 (c.5030G > A, p.(Arg1677Gln)), the causative gene for Coffin-Siris syndrome 6 (CSS6, MIM#617,808) and was inconclusive for the associated episignature due to partial overlap on clustering analysis and a minimally elevated MVP (0.006) (Table 1, Fig. 3). P65 has a VUS in *ZEB2* (c.887A > G, p.(His296Arg)), which matched the episignature for the associated Mowat-Wilson syndrome (MOWS, MIM# 235,730) with moderate confidence (Table 1, Fig. 3). Two patients carrying missense variants in *KDM5C* (c.1646 T > C, p.(Met549Thr) in P34 and c.1837G > A, p.(Glu613Lys) in P68) showed an episignature associated with Claes-Jensen type of X-linked syndromic intellectual developmental disorder (MRXSCJ, MIM#300,534).

Among patients with variants in chromatinopathy-associated genes, concordance between the genetic findings and the expected episignature was observed in approximately half of the cases. This finding underscores the variable sensitivity of episignatures across genes and variant types. The presence of a concordant episignature is considered strong functional (PS3) or phenotypic (PP4) evidence supporting variant pathogenicity. Accordingly, for variants initially classified as VUS or LP according to ACMG/AMP criteria, episignature analysis was applied as additional functional or phenotypic evidence to support variant interpretation. The ACMG criteria applied to each variant are reported in Table S2. When a patient’s DNA methylation profile matched a previously described episignature for the corresponding gene, this concordant molecular evidence was considered alongside the ACMG/AMP data to inform potential reclassification to likely pathogenic or pathogenic. Importantly, epigenetic results were never used alone but always interpreted in the context of genetic and clinical data to ensure robust variant assessment. Not all VUSs could be reclassified after episignature analysis. For those variants where the DNA methylation profile did not match a known episignature, no additional evidence was available to support an upgrade, and the original ACMG/AMP classification was maintained.

## Discussion

With the advent of clinical exome sequencing, chromatinopathies have emerged as an expanding group of individually rare conditions showing partial phenotypic overlap. Collectively, they represent a relatively common cause of intellectual disability. 17% (67/400) of our NDD patients exhibited 66 different variants in chromatinopathy genes. 32% (66/ 205) of the detected variants in all the NDD genes were in chromatinopathy genes. VUS were retained and reported in this study. These findings underscore the importance of chromatinopathies in NDDs.

Ankyrin Repeat Domain 11 (ANKRD11) is a transcriptional regulator that modifies histone acetylation and plays an important role in neurodevelopment. *ANKRD11* is one of the frequently mutated genes associated with NDD. Pathogenic variants in *ANKRD11* cause KBGS, which is characterized by NDD, facial dysmorphism and macrodontia of the upper central incisors [24, 25]. SET domain-containing 5 (SETD5) is a chromatin regulator and acts as a lysine methyltransferase. *SETD5* mutations are another common cause of neurodevelopmental disorder known as intellectual developmental disorder autosomal dominant 23 syndrome (MRD23, MIM#615,761). Patients with pathogenic variants in *SETD5* and *ANKRD11* display overlapping phenotypes [26]. Haploinsufficiency of *ANKRD11* and *SETD5* is the most likely mechanism of pathogenicity for KBGS and MRD23, respectively [24, 27]. Apart from individual P2 (*ANKRD11* c.4554del), all other identified individuals with truncating variants in *ANKRD11* and *SETD5* matched the established combined episignatures associated with both syndromes, KBGS_MRD23. Despite the range of MVPs in the positive cases shown in Fig. 2, additional MDS clustering plots and disorder specific secondary episignatures for KBGS and MRD23 were used to add confidence to our final reporting. Additionally, a previously described *ANKRD11* missense variant (c.7535G > A) located at the highly intolerant C-terminal transcriptional repression domain RD2 [28] which regulates protein activity and appropriate degradation was a moderate match to the episignature associated with KBGS_MRD23 [29, 30]. Three variants in *ANKRD11* and *SETD5,* classified as VUS did not show episignatures concordant with KBGS_MRD23 (Table 1).

ANKRD11, SETD5, and NIPBL interact directly with HDAC3 which plays a central role in the chromatinopathy network. This is reflected by a CdLS-like phenotype, characterized by a distinct craniofacial appearance, pre- and postnatal growth retardation, NDD, and limb anomalies, associated with variants in both *SETD5* and *ANKRD11* [31]. However, the most frequent cause of CdLS accounting for approximately 70% of cases are variants in the cohesion regulator NIPBL [32]. We identified two variants in *NIPBL*: a likely pathogenic truncating variant, c.3525del, and a VUS, c.6065A > G. The c.3525del variant could not be tested for episignatures, whereas c.6065A > G did not exhibit any established disorder-specific episignatures.

Genes encoding histone lysine methylases (KMTs) and demethylases (KDMs) are associated with multisystem developmental disorders, such as Wiedemann-Steiner syndrome (WDSTS, MIM #605,130), which has been associated with the RSTS-like phenotype [33], Kabuki syndrome 1 (KS1, MIM#147,920) and Claes-Jensen type of X-linked syndromic intellectual developmental disorder (MRXSCJ, MIM #300,534). These are autosomal dominant and X-linked inherited conditions [34]. Twenty-four percent of our patients (n = 16) were found to have 15 different variants in the KMTs/ KDMs. Fourteen of these individuals were subjected to EpiSign analysis. Four individuals with variants in *KDM5C* showed an episignature associated with MRXSCJ. Episignature analysis allowed reclassification of two *KDM5C* VUS as likely pathogenic. Furthermore, the episignature associated with WDSTS was observed in four patients with *KMT2A* variants. Two patients with a clinical diagnosis of KS exhibited DNA methylation episignatures concordant with KS having variants in *KMT2D*. However, four patients with VUSs (three missense variants and a start-loss variant) in *KMT2B*, *KMT2C* and *KMT2E* did not match any established disorder specific episignature as these underlying genes are not part of the EpiSign V5 test menu (Table S3). As shown in Table S2, these variants were considered VUS with limited evidence.

CSS causative genes such as *ARID1A*, *ARID2, BICRA, SMARCB1, SMARCD1,* and *SMARCA4* are structural components of the SWItch/Sucrose Nonfermentable complex which is a subfamily of ATP-dependent chromatin remodeling complexes. CSS is characterized by dysmorphic features, NDD, and organ system anomalies, such as cardiac, renal, brain, and skeletal malformations including aplasia or hypoplasia of the distal phalanx or nail of the fifth and additional digits [35]. We identified nine variants in CSS causative genes. From these, established episignatures were available for CSS6 and BAFopathy, which includes cases with variants in *ARID1A, ARID1B, SMARCB1, SMARCA4* or *SMARCA2*. The remaining genes do not have an established specific episignature but underwent a full screen. Only truncating variants exhibited established disorder-specific episignatures, namely c.1109dupT in *ARID2* and c.336-2A > G in *SMARCB1*, which showed DNA methylation episignatures concordant with CSS6 and BAFopathy, respectively. P12 with a missense VUS in *ARID2* was inconclusive for the CSS6 signature on EpiSign (Fig. 3). The CSS6 episignature is defined with truncating variants predicted to result in nonsense-mediated decay and hence loss-of-function [36]. It can be hypothesized that the missense variant may result in a milder profile due to its reduced functional impact as a hypomorphic variant. This reduced impact could explain the inconclusive EpiSign result, which in turn may account for the milder clinical presentation observed in P12 compared to classic CSS.

Genes encoding chromodomain-helicase-DNA-binding proteins (CHDs), such as CHD3, CHD4, CHD5 and CHD8 are ATP-dependent chromatin remodeling proteins that cause autosomal dominant NDDs. CHDs play critical roles in the development of the cerebrum, cerebellum, thalamus, hypothalamus and hippocampus. Therefore, mutations in CHDs affect cognitive functions, the integration of sensory information, memory, learning, and motor coordination [37]. Six different CHD variants were identified in seven patients. Interestingly, individual P20 presenting with typical SNIBCPS symptoms and a missense variant in *CHD3* (c.3692G > A), classified as likely pathogenic, exhibited a methylation profile partially overlapping with Arboleda-Tham syndrome (ARTHS, MIM# 616,268), which is caused by pathogenic variants in *KAT6A* (lysine acetyltransferase 6A) [38]. Critical review of *KAT6A* (coding SNVs and indels) using the WES data did not identify any pathogenic variants in P20. We acknowledge that other variant classes, such as CNVs, SVs, or deep intronic changes, may not have been captured. While the EpiSign Knowledge Database does not include additional cases with this specific *CHD3* variant, other individuals with likely pathogenic and pathogenic variants do not show this overlap. However, since there is not a defined SNIBCPS signature on V5 we cannot rule out this overlap is due to a yet unmapped signature.

Additionally, individual P26, which has a likely pathogenic variant c.4728-2A > G in *CHD8*, displayed a previously established episignature associated with intellectual developmental disorder with autism and macrocephaly (IDDAM, MIM# 615,032). Despite P22 having a pathogenic truncating variant in *CHD4* the individual did not match the SIHIWES signature. This discordance can be explained by the current V5 SIHIWES episignature which is defined by missense variants in the ATPase/helicase domain rather than truncating variants as in case P22.

DNA methyltransferase 3A (DNMT3A) is crucial for genome-wide de novo methylation and is associated with reciprocal disorders: Tatton-Brown-Rahman syndrome (TBRS, MIM#615,879) and Heyn-Sproul-Jackson syndrome (HESJAS, MIM#618,724), which are both characterized by impaired intellectual development [39, 40]. Two different variants were detected in *DNMT3A* in two patients from our cohort. Patient P30, with a novel variant (c.897A > C) in this gene showed typical methylation profile characteristic of TBRS. Patient P2 had a previously described nonsense pathogenic variant (c.427C > T), which interestingly did not exhibit the specific episignature for TBRS [41]. Additionally, a de novo pathogenic variant in *ANKRD11* was detected, however, methylation data did not match the established episignature for KBGS_MRD23. Clinically P2 presents a combination of both KBGS and TBRS features. While patients with two variants have been reported positive for multiple episignatures, the SVM classifier is trained against other signatures to increase specificity and represents only a subset of the differentially methylated probes within a cohort of patients. The KBGS_MRD23 and TBRS episignatures share only 3 of about 200 signature specific probes. If we nevertheless consider the broader KBGS and TBRS methylation profiles they show an overlap of differentially methylated probes, however, with opposite directions of methylation changes in KBGS vs. TBRS. One possible hypothesis for the negative results is that the combination of the two variants may alter the global methylation pattern in a different way, compared to patients with only one underlying variant.

*SIN3A* and *POGZ* are other chromatinopathy genes associated with Witteveen-Kolk syndrome (WITKOS MIM# 613,406) and White-Sutton syndrome (WHSUS MIM# 616,364), respectively. Patient P58 with a novel pathogenic nonsense variant (c.3418C > T) in *SIN3A* exhibited WITKOS episignature. Similarly, patient P50 who had a novel likely pathogenic variant in *POGZ* exhibited a moderate match to the WHSUS episignature. This reduced confidence may be related to the patient’s age (6 months) at which the sample was taken. It is well known that age can influence global methylation profiles.

PHD finger-containing protein 1 (BRPF1) acts as a histone H3 acetyltransferase, and its mutations are responsible for intellectual developmental disorder with dysmorphic facies and ptosis [42]. We have a single patient (P18) with a likely pathogenic variant in *BRPF1* (c.2972 T > G) who underwent a full screen but did not match any known episignatures, likely due to the absence of a defined signature for this gene.

*BPTF* encodes for bromodomain PHD finger transcription factor, which is the largest subunit of nucleosome remodeling factor (NURF), a member of the ISWI chromatin-remodeling complex. Individual P17 was found to have a c.613 + 1del variant in *BPTF*, showing a distinct methylation profile partially overlapping with the MRD21 episignature. This profile may be explained by the functional and physical connections between NURF chromatin remodeling and the ubiquitous and multivalent regulators *CTCF* (associated with Intellectual developmental disorder, autosomal dominant 21, MRD21, MIM# 615,502) and the cohesion complex (associated with CdLS) during gene expression [43]. Since episignatures are created with probes that represent global methylation profiles it can be hypothesized that this overlap may be due to a shared global profile due to the disruption of this protein interaction caused by the *BPTF* variant. Our findings provide preliminary evidence for a distinct DNA methylation profile associated with pathogenic variants in *BPTF*. At present, this profile should be considered a candidate episignature, as confirmation will require larger reference cohorts, classifier training, and independent replication. This highlights the ability for EpiSign to identify cases that share functional pathways and supports a search for underlying variants extending beyond the genes used to define a signature.

Several individuals carrying pathogenic or likely pathogenic variants did not exhibit the expected episignature. This may reflect allelic heterogeneity, variant-specific effects, incomplete penetrance of epigenetic alterations, or limitations in the current reference episignature datasets.

Interpretation of episignature results in this study follows recently published expert consensus recommendations developed by the EpiSign Clinical Testing Network [8]. These guidelines were established by a clinical working group with extensive experience in episignature analysis and reporting, with the aim of harmonizing interpretation practices across multiple health jurisdictions, including the United Kingdom, United States, and European Union. The recommendations define standardized reporting categories based on the presence or absence of a disease-specific DNA methylation episignature in the context of an associated gene variant, as summarized in Table 3 of the consensus manuscript [8]. Within this framework, the presence of a concordant episignature is considered strong functional (PS3) or phenotypic (PP4) evidence supporting variant pathogenicity and may be sufficient to support reclassification of a variant of uncertain significance to likely pathogenic. In contrast, the absence of an episignature is regarded as supportive—but not conclusive—evidence against pathogenicity.

Applying this consensus-based framework, the approximately 50% rate of episignature support observed in our chromatinopathy-enriched cohort is consistent with prior clinical experience and compares favorably with previously published targeted EpiSign study, which reported positivity rates of approximately 35% when testing was primarily ordered for VUS resolution [8]. The detectability of an episignature is influenced by several factors, including variant type, gene-specific disease mechanisms, and the magnitude of epigenetic disruption measurable in peripheral blood. Heterozygous variants—particularly those classified as VUS—may result in partial loss of function, variable penetrance, or context-dependent effects that do not uniformly produce a robust DNA methylation signature. Taken together, these findings reinforce the role of episignature analysis as a complementary functional assay rather than a standalone diagnostic test. Its greatest value lies in providing strong supportive evidence when positive, while negative results must be interpreted cautiously within an integrated genomic and clinical context.

In this study, genome-wide DNA methylation analysis provided evidence to reclassify 10 LP variants as P and three VUS as LP. Reclassification was not possible for the remaining 24 VUS variants. Importantly, according to current recommendations for interpreting EpiSign results, a negative or inconclusive episignature should not be taken as evidence against pathogenicity. Instead, such findings should be evaluated in the context of clinical presentation [8]. Twenty-one of the chromatinopathy variants identified are not detectable in the EpiSign V5 test menu and additional discovery work remains for establishing specific episignatures and downstream testing for any of these VUS cases. Discordant or absent episignatures should be interpreted with caution, as they may reflect pathogenic variants not detectable by whole-exome sequencing, such as CNVs, SVs, or non-coding variants. Therefore, we emphasize that episignature discordance should prompt consideration of complementary approaches including genome sequencing and comprehensive CNV and SV analysis. In this context, EpiSign analysis is not a substitute for comprehensive genomic testing but rather a complementary tool that can enhance diagnostic yield, refine variant interpretation, highlight diagnostic inconsistencies, and guide subsequent testing strategies. Importantly, this study was not designed as a comprehensive genome-wide diagnostic yield assessment. Instead, it represents a retrospective, WES-anchored cohort enriched for chromatinopathy gene findings, with episignature analysis applied as a secondary functional tool. These considerations underscore the need to interpret episignature results within an integrated diagnostic framework that includes complementary genomic approaches when clinically indicated.

Episignature analysis can be integrated into the ACMG/AMP framework as supporting evidence. Concordance between a patient’s methylation profile and a gene-specific episignature may reinforce the functional impact of a variant, similar to functional assays or strong phenotype correlations. However, epigenetic data should be interpreted in combination with standard ACMG criteria, and not as an independent criterion, to guide robust variant reclassification. P17 highlights the utility of a full screen for cases regardless of variants identified and that a partially overlapping methylation profile may actually showcase relevant functional overlap with a variant in a different underlying gene contributing to the methylation profile.

## Conclusion

In the current study, 17 percent of our patients with a clinical NDD diagnosis harbored variants in chromatinopathy genes. We identified 55 novel variants (31 LP/P variants, 24 VUS) in genes encoding chromatin remodelers and transcriptional regulators, thus, expanding the genotypic spectrum of chromatinopathies. Close to fifty percent of the tested individuals matched previously established episignatures. As the number of established episignatures is continually expanding, future reanalysis of the methylation data has the potential to identify a relevant episignature, thereby, allowing reclassification especially of VUS.

## Supplementary Information

Additional file1

Additional file2

Additional file3

Additional file4

## Data Availability

No datasets were generated or analysed during the current study.

## Abbreviations

ARTHS: Arboleda-Tham syndrome
BRPF1: PHD finger-containing protein 1
CdLS: Cornelia de Lange syndrome
CHD: Chromodomain-helicase-DNA-binding protein
CSS: Coffin-Siris syndrome
DNMT3A: DNA methyltransferase 3A
HESJAS: Heyn-Sproul-Jackson syndrome HESJAS
HGMD: Human gene mutation database
IDDAM: Intellectual developmental disorder with autism and macrocephaly
KBGS: KBG syndrome
KDM: Histone lysine demethylase
KMT: Histone lysine methylase
LOVD: Leiden open variation database
LP: Likely pathogenic
MOWS: Mowat-Wilson syndrome
MRD21: Intellectual developmental disorder, autosomal dominant 21
MRD23: Intellectual developmental disorder autosomal dominant 23
MRXSCJ: Claes-Jensen type of X-linked syndromic intellectual developmental disorder
MVP: Methylation variant pathogenicity
NDD: Neurodevelopmental disorder
NGS: Next generation sequencing
P: Pathogenic
RSTS: Rubinstein-Taybi syndrome
SIHIWES: Sifrim-Hitz-Weiss syndrome
SVM: Support vector machine
TBRS: Tatton-Brown-Rahman syndrome
VUS: Variants of uncertain significance
WES: Whole-exome sequencing
WDSTS: Wiedemann-Steiner syndrome
WITKOS: Witteveen-Kolk syndrome
WHSUS: White-Sutton syndrome
