# Correction: Chromatinopathies: clinically overlapping disorders, revealing novel variants and their DNA methylation signatures

**DOI:** 10.1186/s13148-026-02173-2

**Published:** 2026-06-13

**Authors:** Asuman Koparir, Jennifer Kerkhof, Jessica Rzasa, Eva Metzger, Paulina Bahena Carbajal, Konstantinos Kolokotronis, Erkan Koparir, Yvonne Jelting, Michaela A. H. Hofrichter, Jörg Klepper, Thomas König, Eva Runkel, Wahyu Eka Prastyo, Jonas Deinlein, Neda Dragicevic Babic, Juliane Spiegler, Nicole Stachelscheid, Erdmute Kunstmann, Thomas Haaf, Bekim Sadikovic, Eva Klopocki

**Affiliations:** 1 Institute of Human Genetics, Julius Maximilians University Würzburg, Würzburg, Germany; 2 Verspeeten Clinical Genome Centre, London Health Sciences Centre, London, ON Canada; 3 MVZ Genetikum GmbH, Neu-Ulm, Germany; 4 Institute of Medical Genetics, University Hospital Rostock, Rostock, Germany; 5 Institute of Medical Genetics, University of Zurich, Zurich, Swiss Confederation Switzerland; 6 Center of Human Genetics, Tübingen, Germany; 7 Department of Pediatrics and Neuropediatrics, Klinikum Aschaffenburg- Alzenau, Aschaffenburg, Germany; 8 Department of Pediatrics, University Hospital Würzburg, Würzburg, Germany; 9 Present Address: Institute of Clinical Genetics and Genomic Medicine, University Hospital Würzburg, Würzburg, Germany; 10 Department of Pathology and Laboratory Medicine, Western University, London, ON Canada

**Correction to: Clin Epigenet (2026) 18:69**

10.1186/s13148-026-02120-1

In this article [1], The Table 1, the values in P62, gene SMARCB1 (NM_003073.5) was incorrectly published as c.336–2 A > G instead of c.363–2 A > G

Incorrect Table 1 Value:

**Table:**
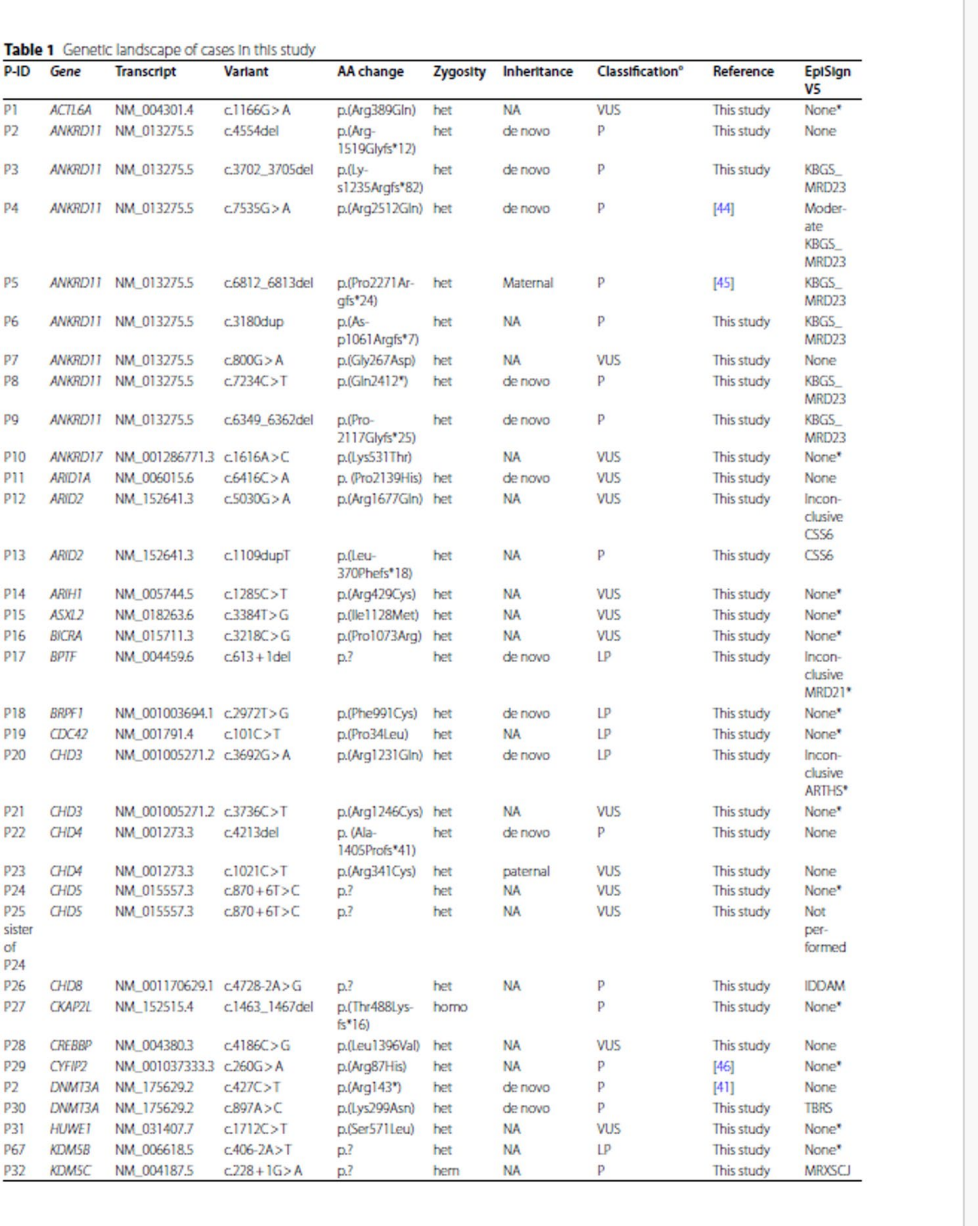

**Table:**
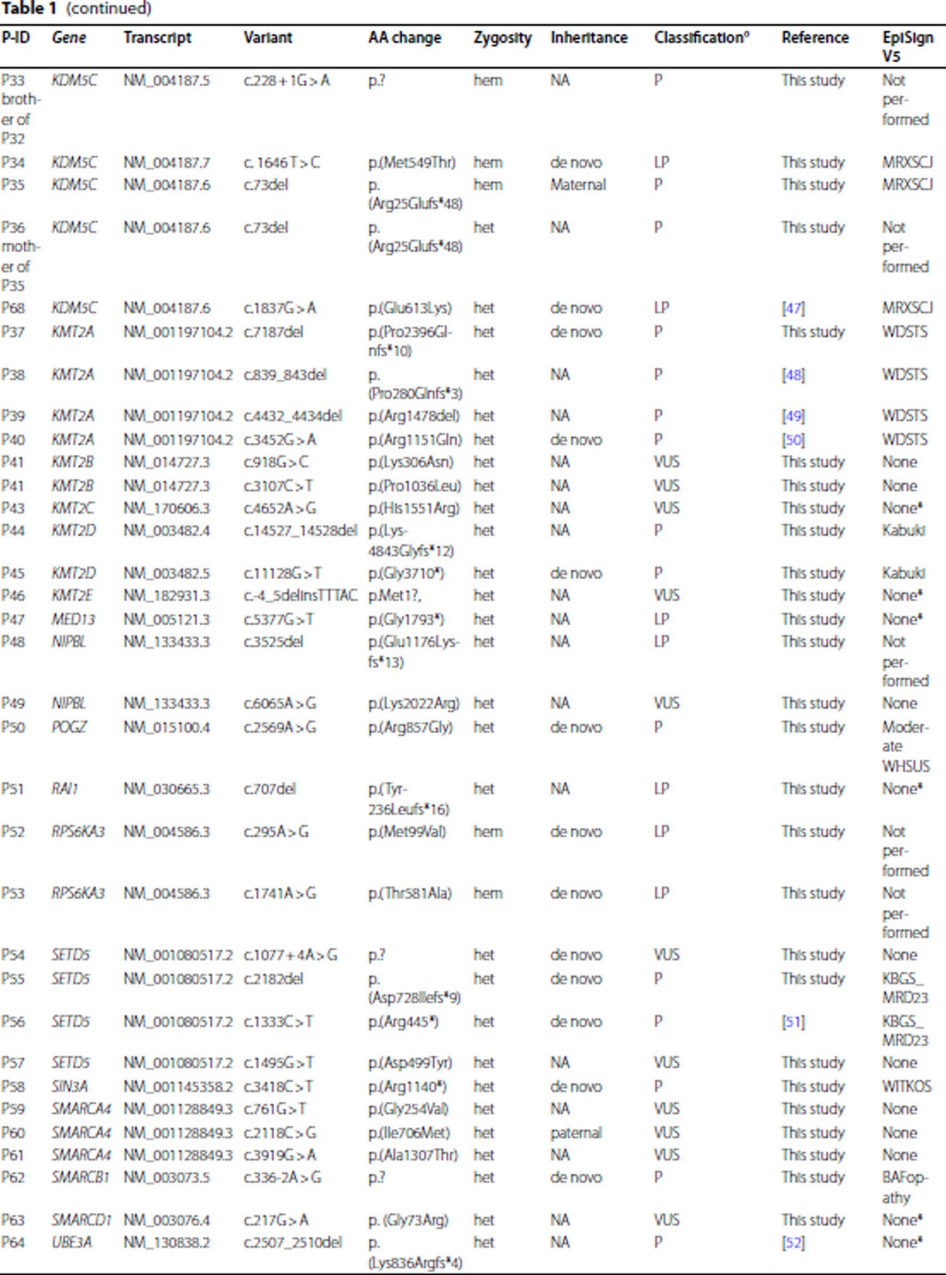

**Table:**
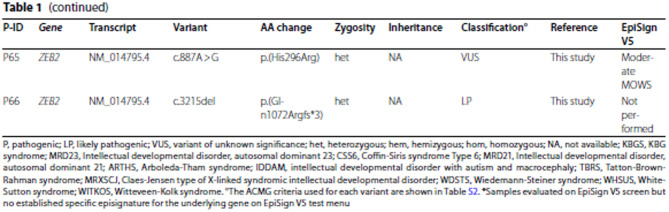

Correct Table 1 Value:

**Table 1:**
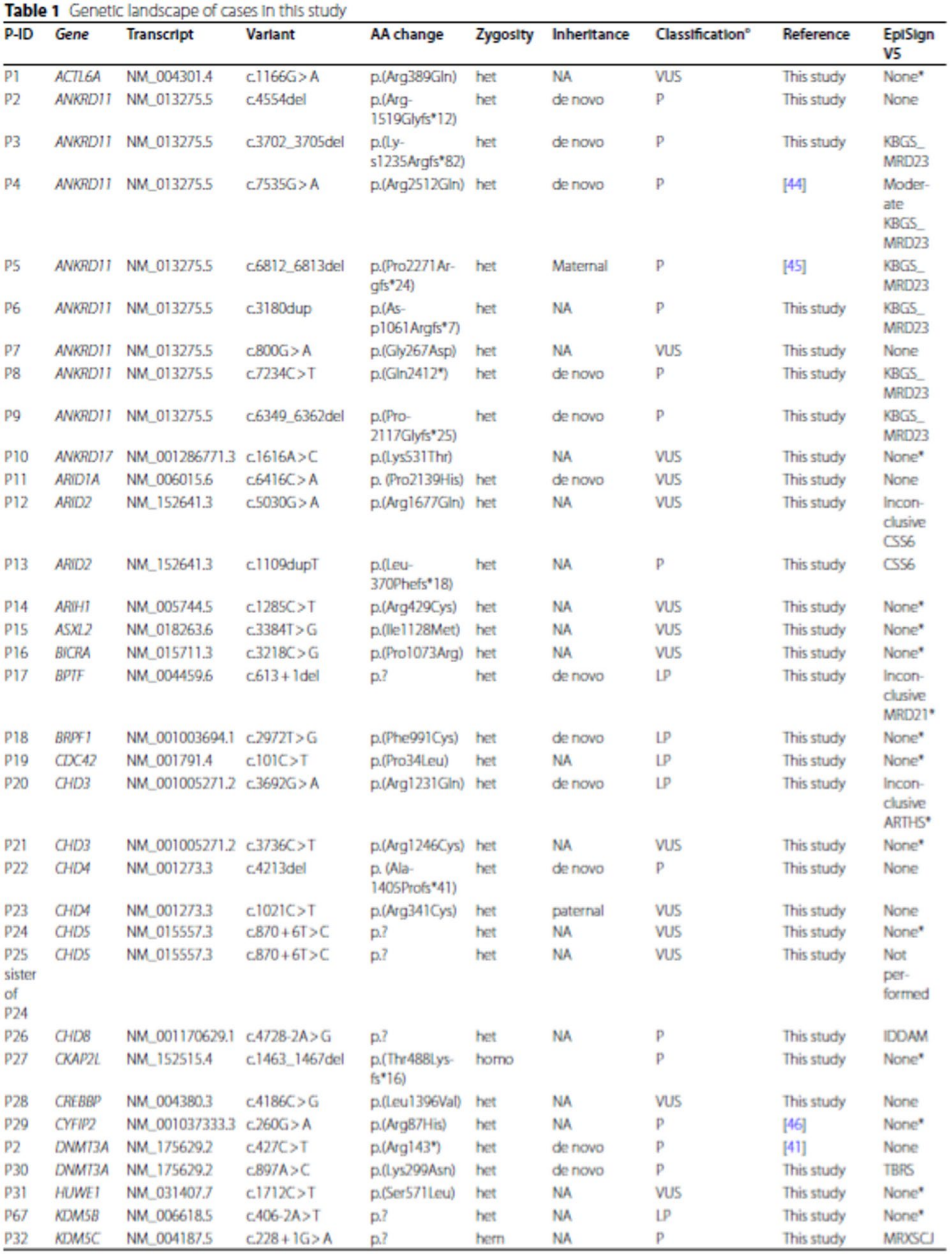

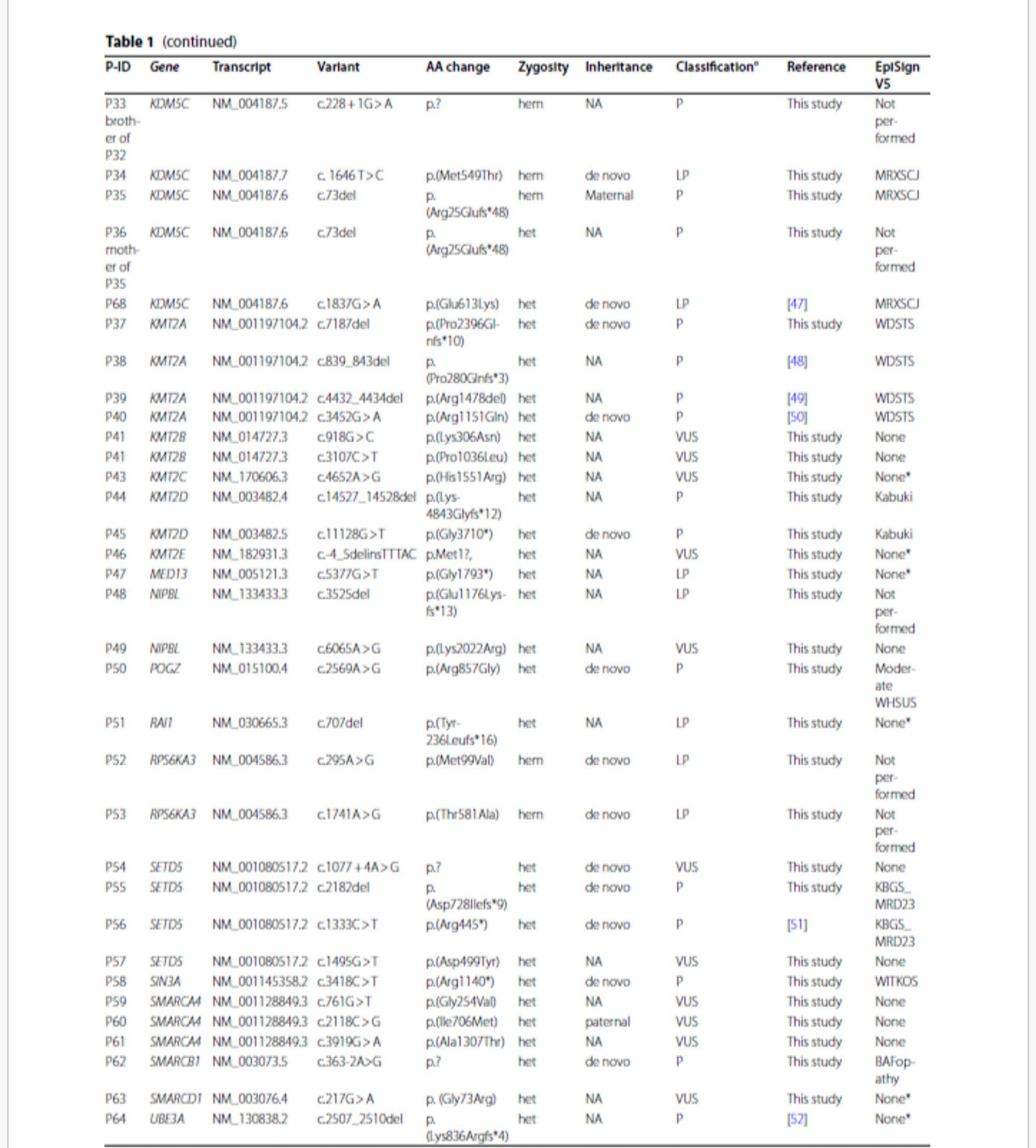

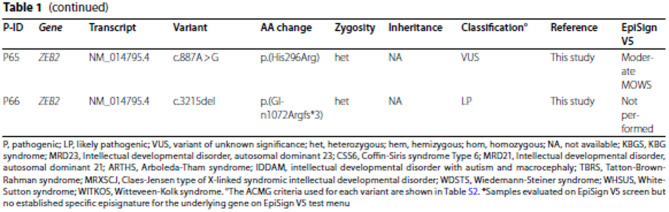

## References

[CR1] 1. Koparir, A., Kerkhof, J., Rzasa, J. *et al.* Chromatinopathies: clinically overlapping disorders, revealing novel variants and their DNA methylation signatures. *Clin Epigenet* **18**, 69 (2026) 10.1186/s13148-026-02120-1 PMC13088768 41957673

[CR2] The original article can be found online at 10.1186/s13148-026-02120-1

