# Supplementary figures and images for: Chromatinopathies: clinically overlapping disorders, revealing novel variants and their DNA methylation signatures
Source: Clin Epigenetics. 2026 Apr 9;18:69. doi: 10.1186/s13148-026-02120-1 (PMC13088768; doi:10.1186/s13148-026-02120-1)

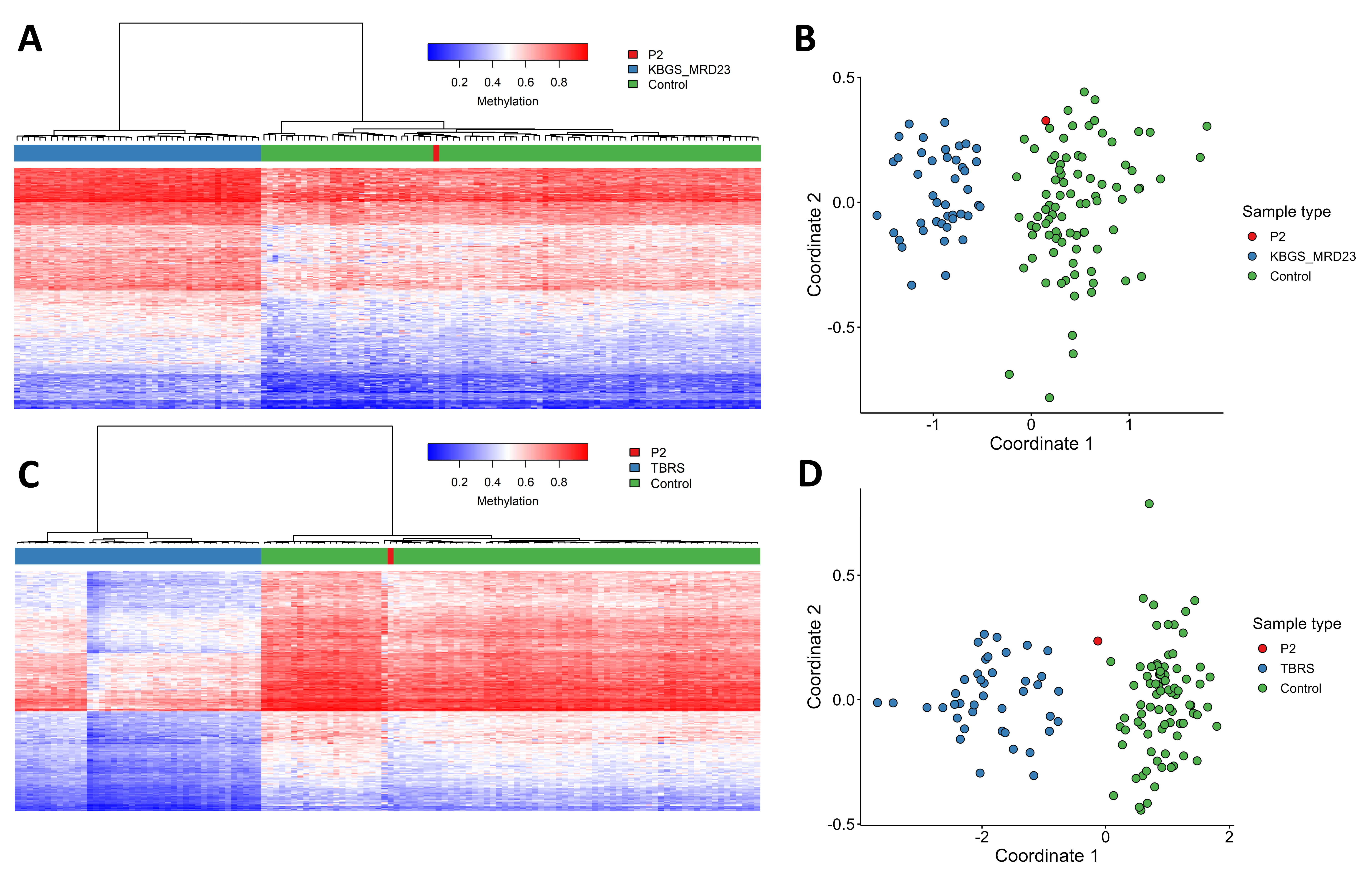

Supplement: Supplementary file 1 — Additional file1 [file 13148_2026_2120_MOESM1_ESM.tif]
