# Supplementary material for: Chromatinopathies: clinically overlapping disorders, revealing novel variants and their DNA methylation signatures
Source: Clin Epigenetics. 2026 Apr 9;18:69. doi: 10.1186/s13148-026-02120-1 (PMC13088768; doi:10.1186/s13148-026-02120-1)
Supplement: Supplementary file 2 — Additional file2 [file 13148_2026_2120_MOESM2_ESM.docx]

| **P-ID** | **Age in years*** | **Sex** |
| --- | --- | --- |
| P1 | 17 | Male |
| P2 | 12 | Male |
| P3 | 3 | Male |
| P4 | 15 | Female |
| P5 | 2 | Male |
| P6 | 2 | Female |
| P7 | 4 | Male |
| P8 | 9 | Male |
| P9 | 27 | Female |
| P10 | 2 | Male |
| P11 | 4 | Female |
| P12 | 3 | Male |
| P13 | 3 | Male |
| P14 | 5 | Male |
| P15 | 6 | Male |
| P16 | 8 | Male |
| P17 | 6 | Female |
| P18 | 6 | Female |
| P19 | 5 | Female |
| P20 | 33 | Female |
| P21 | 20 | Female |
| P22 | 6 | Female |
| P23 | 3 | Male |
| P24 | 3 | Female |
| P25 sister of P24 | 4 | Female |
| P26 | 7 | Female |
| P27 | 64 | Male |
| P28 | 1 | Female |
| P29 | 19 | Male |
| P30 | 3 | Female |
| P31 | 7 | Male |
| P32 | 40 | Male |
| P33 brother of P32 | 36 | Male |
| P34 | 10 | Male |
| P35 | 6 | Male |
| P36 mother of P35 | 43 | Female |
| P37 | 6 | Female |
| P38 | 8 | Male |
| P39 | 2 | Female |
| P40 | 1 | Female |
| P41 | 5 | Female |
| P43 | 2 | Female |
| P44 | 6 | Male |
| P45 | 2 | Male |
| P46 | 5 | Female |
| P47 | 5 | Male |
| P48 | 0,5 | Male |
| P49 | 1 | Male |
| P50 | 0,5 | Male |
| P51 | 12 | Male |
| P52 | 5 | Male |
| P53 | 7 | Male |
| P54 | 6 | Female |
| P55 | 2 | Male |
| P56 | 7 | Female |
| P57 | 8 | Male |
| P58 | 4 | Male |
| P59 | 5 | Female |
| P60 | 8 | Male |
| P61 | 7 | Male |
| P62 | 3 | Female |
| P63 | 5 | Female |
| P64 | 2 | Female |
| P65 | 4 | Male |
| P66 | 61 | Female |
| P67 | 7 | Male |
| P68 | 8 | Female |

* at the time of sample collection
