# Supplementary material for: Chromatinopathies: clinically overlapping disorders, revealing novel variants and their DNA methylation signatures
Source: Clin Epigenetics. 2026 Apr 9;18:69. doi: 10.1186/s13148-026-02120-1 (PMC13088768; doi:10.1186/s13148-026-02120-1)
Supplement: Supplementary file 3 — Additional file3 [file 13148_2026_2120_MOESM3_ESM.docx]

| **Gene** | **Transcript** | **Variant** | **AA change** | **ACMG Criteria** | **Classification** | **EpiSign V5** | **Classification after EpiSign** |
| --- | --- | --- | --- | --- | --- | --- | --- |
| *ACTL6A* | NM_004301.4 | c.1166G>A | p.(Arg389Gln) | PM2_Sup, PP3_Mod | VUS | None* | VUS |
| *ANKRD11* | NM_013275.5 | c.4554del | p.(Arg1519Glyfs*12) | PVS1, PM2_Sup, PM6 | P | None | P |
| *ANKRD11* | NM_013275.5 | c.3702_3705del | p.(Lys1235Argfs*82) | PVS1, PM2_Sup, PM6 | P | KBGS_MRD23 | P |
| *ANKRD11* | NM_013275.5 | c.7535G>A | p.(Arg2512Gln) | PS3, PS4_Sup, PM2_Sup, PM6,  PP3_Sup | P | Moderate KBGS_MRD23 | P |
| *ANKRD11* | NM_013275.5 | c.6812_6813del | p.(Pro2271Argfs*24) | PVS1, PS4_Sup, PM2_Sup | P | KBGS_MRD23 | P |
| *ANKRD11* | NM_013275.5 | c.3180dup | p.(Asp1061Argfs*7) | PVS1, PM2_Sup | LP | KBGS_MRD23 | P |
| *ANKRD11* | NM_013275.5 | c.800G>A | p.(Gly267Asp) | PM2_Sup, PP3_Sup | VUS | None | VUS |
| *ANKRD11* | NM_013275.5 | c.7234C>T | p.(Gln2412*) | PVS1, PM2_Sup, PM6 | P | KBGS_MRD23 | P |
| *ANKRD11* | NM_013275.5 | c.6349_6362del | p.(Pro2117Glyfs*25) | PVS1, PM2_Sup, PM6 | P | KBGS_MRD23 | P |
| *ANKRD17* | NM_001286771.3 | c.1616A>C | p.(Lys531Thr) | PM2_Sup, PP3_Sup | VUS | None* | VUS |
| *ARID1A* | NM_006015.6 | c.6416C>A | p.(Pro2139His) | PM2_Sup, PP3_Sup, PM6 | VUS | None | VUS |
| *ARID2* | NM_152641.3 | c.5030G>A | p.(Arg1677Gln) | PM2_Sup, PP3_Sup,  PP2 | VUS | Inconclusive CSS6 | VUS |
| *ARID2* | NM_152641.3 | c.1109dupT | p.(Leu370Phefs*18) | PVS1, PM2_Sup | LP | CSS6 | P |
| *ARIH1* | NM_005744.5 | c.1285C>T | p.(Arg429Cys) | PM2_Sup,  PP3_Mod | VUS | None* | VUS |
| *ASXL2* | NM_018263.6 | c.3384T>G | p.(Ile1128Met) | PM2_Sup | VUS | None* | VUS |
| *BICRA* | NM_015711.3 | c.3218C>G | p.(Pro1073Arg) | PM2_Sup | VUS | None* | VUS |
| *BPTF* | NM_004459.6 | c.613+1del | p.? | PVS1_Strong, PM2_Sup, PM6 | LP | Inconclusive MRD21* | LP |
| *BRPF1* | NM_001003694.1 | c.2972T>G | p.(Phe991Cys) | PM2_Sup,  PP3_Sup,  PP2  PP4  PM6 | LP | None* | LP |
| *CDC42* | NM_001791.4 | c.101C>T | p.(Pro34Leu) | PS1, PM2_Sup,  PP3_Sup, | LP | None* | LP |
| *CHD3* | NM_001005271.2 | c.3692G>A | p.(Arg1231Gln) | PM1,  PM2_Sup  PP3_Mod  PM6 | LP | Inconclusive ARTHS* | LP |
| *CHD3* | NM_001005271.2 | c.3736C>T | p.(Arg1246Cys) | PM1,  PM2_Sup  PP3_Sup | VUS | None* | VUS |
| *CHD4* | NM_001273.3 | c.4213del | p.(Ala1405Profs*41) | PVS1,  PM2_Sup,  PM6 | P | None | P |
| *CHD4* | NM_001273.3 | c.1021C>T | p.(Arg341Cys) | PM2_Sup  PP3_Sup | VUS | None | VUS |
| *CHD5* | NM_015557.3 | c.870+6T>C | p.? | PM2_Sup | VUS | None* | VUS |
| *CHD8* | NM_001170629.1 | c.4728-2A>G | p.? | PVS1,  PM2_Sup | LP | IDDAM | P |
| *CKAP2L* | NM_152515.4 | c.1463_1467del | p.(Thr488Lysfs*16) | PVS1,  PS4-Sup,  PM2_Sup | P | None* | P |
| *CREBBP* | NM_004380.3 | c.4186C>G | p.(Leu1396Val) | PM2_Sup  PP3_Sup | VUS | None | VUS |
| *CYFIP2* | NM_001037333.3 | c.260G>A | p.(Arg87His) | PS1,  PS4_Mod,  PM1,  PM2_Sup  PP3_Mod | P | None* | P |
| *DNMT3A* | NM_175629.2 | c.427C>T | p.(Arg143*) | PVS1,  PS4_Sup  PM2_Sup | P | None | P |
| *DNMT3A* | NM_175629.2 | c.897A>C | p.(Lys299Asn) | PS1,  PM2_Sup  PP3_Sup  PM6 | LP | TBRS | P |
| *HUWE1* | NM_031407.7 | c.1712C>T | p.(Ser571Leu) | PM2_Sup  PP3_Sup | VUS | None* | VUS |
| *KDM5B* | NM_006618.5 | c.406-2A>T | p.? | PVS1,  PM2_Sup | LP | None* | LP |
| *KDM5C* | NM_004187.5 | c.228+1G>A | p.? | PVS1,  PM2_Sup | LP | MRXSCJ | P |
| *KDM5C* | NM_004187.7 | c. 1646T>C | p.(Met549Thr) | PM2_Sup  PP3_Mod  PM6 | VUS | MRXSCJ | LP |
| *KDM5C* | NM_004187.6 | c.73del | p.(Arg25Glufs*48) | PVS1,  PM2_Sup | LP | MRXSCJ | P |
| *KDM5C* | NM_004187.6 | c.1837G>A | p.(Glu613Lys) | PS4_Sup  PM2_Sup  PP3_Sup  PM6 | VUS | MRXSCJ | LP |
| *KMT2A* | NM_001197104.2 | c.7187del | p.(Pro2396Glnfs*10) | PVS1,  PM2_Sup,  PM6 | P | WDSTS | P |
| *KMT2A* | NM_001197104.2 | c.839_843del | p.(Pro280Glnfs*3) | PVS1,  PS4_Sup  PM2_Sup | P | WDSTS | P |
| *KMT2A* | NM_001197104.2 | c.4432_4434del | p.(Arg1478del) | PVS1_Strong,  PS4_Sup  PM2_Sup | LP | WDSTS | P |
| *KMT2A* | NM_001197104.2 | c.3452G>A | p.(Arg1151Gln) | PS4_Sup  PM1  PM2_Sup  PP3_Sup  PM6 | LP | WDSTS | P |
| *KMT2B* | NM_014727.3 | c.918G>C | p.(Lys306Asn) | PM2_Sup | VUS | None | VUS |
| *KMT2B* | NM_014727.3 | c.3107C>T | p.(Pro1036Leu) | PM2_Sup | VUS | None | VUS |
| *KMT2C* | NM_170606.3 | c.4652A>G | p.(His1551Arg) | PM2_Sup | VUS | None* | VUS |
| *KMT2D* | NM_003482.4 | c.14527_14528del | p.(Lys4843Glyfs*12) | PVS1,  PM2_Sup | LP | Kabuki | P |
| *KMT2D* | NM_003482.5 | c.11128G>T | p.(Gly3710*) | PVS1,  PM2_Sup  PM6 | P | Kabuki | P |
| *KMT2E* | NM_182931.3 | c.-4_5delinsTTTAC | p.Met1?, | PM2_Sup | VUS | None* | VUS |
| *MED13* | NM_005121.3 | c.5377G>T | p.(Gly1793*) | PVS1,  PM2_Sup | LP | None* | LP |
| *NIPBL* | NM_133433.3 | c.3525del | p.(Glu1176Lysfs*13) | PVS1,  PM2_Sup | LP | Not performed | Not performed |
| *NIPBL* | NM_133433.3 | c.6065A>G | p.(Lys2022Arg) | PM2_Sup | VUS | None | VUS |
| *POGZ* | NM_015100.4 | c.2569A>G | p.(Arg857Gly) | PS2,  PM2_Sup,  PP3_Mod | LP | Moderate WHSUS | P |
| *RAI1* | NM_030665.3 | c.707del | p.(Tyr236Leufs*16) | PVS1,  PM2_Sup | LP | None* | LP |
| *RPS6KA3* | NM_004586.3 | c.295A>G | p.(Met99Val) | PM2_Sup  PM6  PP3_Sup  PP4 | LP | Not performed | Not performed |
| *RPS6KA3* | NM_004586.3 | c.1741A>G | p.(Thr581Ala) | PS4_Sup  PM2_Sup  PM6  PP3_Mod  PP4 | LP | Not performed | Not performed |
| *SETD5* | NM_001080517.2 | c.1077+4A>G | p.? | PM2_Sup  PP3_Sup | VUS | None | VUS |
| *SETD5* | NM_001080517.2 | c.2182del | p.(Asp728Ilefs*9) | PVS1,  PM2_Sup  PM6 | P | KBGS_MRD23 | P |
| *SETD5* | NM_001080517.2 | c.1333C>T | p.(Arg445*) | PVS1,  PM2_Sup  PM6 | P | KBGS_MRD23 | P |
| *SETD5* | NM_001080517.2 | c.1495G>T | p.(Asp499Tyr) | PM2_Sup | VUS | None | VUS |
| *SIN3A* | NM_001145358.2 | c.3418C>T | p.(Arg1140*) | PVS1,  PM2_Sup  PM6 | P | WITKOS | P |
| *SMARCA4* | NM_001128849.3 | c.761G>T | p.(Gly254Val) | PM2_Sup  PP3_Mod | VUS | None | VUS |
| *SMARCA4* | NM_001128849.3 | c.2118C>G | p.(Ile706Met) | PM2_Sup  PP3_Sup | VUS | None | VUS |
| *SMARCA4* | NM_001128849.3 | c.3919G>A | p.(Ala1307Thr) | PM2_Sup  PP3_Mod | VUS | None | VUS |
| *SMARCB1* | NM_003073.5 | c.363-2A>G | p.? | PVS1,  PM2_Sup,  PM6 | P | BAFopathy | P |
| *SMARCD1* | NM_003076.4 | c.217G>A | p.(Gly73Arg) | PM2_Sup  PP3_Sup | VUS | None* | VUS |
| *UBE3A* | NM_130838.2 | c.2507_2510del | p.(Lys836Argfs*4) | PVS1,  PS4_Sup,  PM2_Sup | P | None* | P |
| *ZEB2* | NM_014795.4 | c.887A>G | p.(His296Arg) | PM1,  PM2_Sup,  PP2,  PP3_Sup | VUS | Moderate MOWS | LP |
| *ZEB2* | NM_014795.4 | c.3215del | p.(Gln1072Argfs*3) | PVS1,  PM2_Sup | LP | Not performed | Not performed |

Abbreviations: Sup, supporting; Mod, moderate; P, pathogenic; LP, likely pathogenic; VUS, variant of unknown significance. * Samples evaluated on EpiSign V5

screen but no established specific episignature for the underlying gene on EpiSign V5 test menu.
